# Therapeutic efficacy and safety of botulinum toxin type A in trigeminal neuralgia: a systematic review

**DOI:** 10.1186/1129-2377-14-72

**Published:** 2013-08-21

**Authors:** Yong Hu, Xiaofei Guan, Lin Fan, Mu Li, Yiteng Liao, Zhiyu Nie, Lingjing Jin

**Affiliations:** 1 Department of neurology, Shanghai Tongji Hospital, Tongji University School of Medicine, Xin-Cun Road 389, Shanghai 200065, China

**Keywords:** Botulinum toxin, Trigeminal neuralgia, Systematic review, Therapy

## Abstract

Trigeminal neuralgia is a common disorder caused mainly by compression of the trigeminal nerve root by an overlying blood vessel. Pharmacotherapy and surgery are ineffective or unsuitable in many patients. Therefore, other therapeutic modalities have been tried, including injection of botulinum toxin type A (BTX-A). This study aims to systematically review the therapeutic efficacy and safety of BTX-A in trigeminal neuralgia. PubMed, EMBASE, Cochrane Library Clinical Trials and Web of Science from January 1966 to March 2013 were searched with the terms of “botulinum toxin” AND “trigeminal neuralgia”, and references of related articles were traced. Data on the efficacy and safety of BTX-A in this disorder were extracted and analyzed by at least 2 reviewers. Data for individual studies were reported, and pooled data were analyzed if appropriate. Five prospective studies and one double-blind, randomized, placebo-controlled study were identified. Response was achieved in approximately 70-100% of patients, and the mean pain intensity and frequency were reduced by approximately 60-100% at 4 weeks after treatment in most studies. Major adverse events were not reported. Available studies show BTX-A may be effective in treatment of trigeminal neuralgia. However, well-designed randomized, controlled, double-blinded trial is still lacking. Future BTX-A treatment studies on optimal dose, duration of the therapeutic efficacy, common AEs, and the time and indications for repeat injection would be promising.

## Review

### Introduction

Trigeminal neuralgia is a unilateral disorder characterized by brief electric shock-like pains, abrupt in onset and termination, limited to the distribution of one or more divisions of the trigeminal nerve [1]. Epidemiological studies reveal that approximately 4-28.9/100,000 individuals worldwide experience TN [2-5]. It is the most widely recognized neuropathic pain of the face and has been shown to be profoundly distressing the patient’s well-being [6]. TN frequently occurs in subjects aged 50-70 years and is more common in women [2,7]. Compression of the trigeminal nerve near the dorsal root entry zone [8-12] by an overlaying blood vessel is a major causative or contributing factor [8]. In addition, it can also be caused by tumor, multiple sclerosis [13,14], infiltration, amyloid [15-18], small infarcts or angiomas in the pons or medulla [19-21]. In a small fraction of patients, the cause of TN cannot be identified [22].

The treatment of TN continues to be a major challenge due to the complexity of TN’s causes and the trigeminal nerve. The antiepileptic drugs, such as carbamazepine [23,24] oxcarbazepine [25,26] and phenytoin [27,28], are commonly used in the treatment of TN, but a substantial proportion of patients have poor response to this treatment, predominantly because of their side effects related to the central nervous system [6]. Eventually, many TN patients become refractory to antiepileptic drugs and other drugs [29-32]. The quality of evidence on the efficacy of neurosurgical procedures (such as percutaneous interventions of the Gasserian ganglion, stereotactic radiosurgery or microvascular decompression [33]) is very low. Although these procedures may relieve the pain to different extents, many may result in sensory side effects.

Botulinum toxin type A (BTX-A), one of the seven antigenically different botulinum neurotoxins derived from *Clostridium botulinum*, appears to be the most potent subtype [34]. It can cleave the synaptosome-associated protein of 25 kDa (SNAP-25) in the motor nerve terminals [35,36]. BTX-A is reported to be effective in the treatment of migraine and myofacial pain syndrome [37-40]. The mechanism of potential analgesic effect of BTX-A is still unclear. In vitro studies have shown that BTX-A can inhibit the release of pro-inflammatory neuropeptides. Animal experiments also reveal the antinociceptive effect of BTX-A in both inflammatory and neuropathic pain models [41-47]. In 2002, Micheli et al reported the successful treatment of a patient with hemifacial spasm associated with TN with onabotulinumtoxin A, which opens up new possibilities for its use [48]. After that, several other open-label trials have examined the preventive effects of BTX-A on TN [49-51].

The current review is to systematically review the therapeutic efficacy of BTX-A in TN. The secondary goal of this review was to address the safety and tolerability of BTX-A in the treatment of TN.

### Methods

The methodology utilized in this review followed the review process derived from evidence-based systematic reviews and meta-analyses [52-55] of clinic trials and semi-trials.

#### Literature search

A comprehensive search was conducted from 1966 to 2012 using databases including PubMed, EMBASE (OVID), Cochrane Library Clinical Trials and Web of Science. The PubMed, search was conducted by using combinations of Medical Subject Heading (MeSH) search terms and keywords according to the following algorithm: (((“Trigeminal Neuralgia”[Mesh]) OR ((trigeminal [All Fields]) AND neuralgia [All Fields]))) AND (((“Botulinum Toxins, Type A”[Mesh] OR “Botulinum Toxins”[Mesh])) OR botuli* [All Fields]). Other databases were queried by using identical terms for keyword searching. The cross-referencing of bibliographies from notable primary and review articles, and abstracts from scientific meetings and peer-reviewed non-indexed journals were also searched. Only English articles were collected.

At least 2 authors independently, in an unblinded standardized manner, performed searching. Any disagreements were resolved by a third author.

#### Criteria for inclusion of studies for review

All studies were reviewed by at least 2 reviewers for inclusion. Any disagreements were resolved by a third author. If there was a conflict of interest with the reviewed manuscripts with authorship, the involved authors did not review the manuscripts.

##### Types of studies

Randomized controlled trials, semi-trials (case–control studies, open-label studies and case series studies) were selected for evaluation the efficacy and/or safety of BTX in the treatment of TN. When the selected articles reported the same trial, only the latest study with the largest sample size or longest follow-up period was included.

Articles having no original data (such as letters, editorials, commentaries and reviews) and those without adequate information regarding the outcome were excluded. Nonhuman studies were also excluded.

##### Types of participants

Patients with TN of all ages, sex, and degrees of severity were included. TN was diagnosed according to the criteria developed by the International Headache Society (IHS) or other criteria that conformed in general to the IHS diagnostic criteria [1].

##### Types of interventions

Included studies had to use either a single dose of BTX-A to treat TN, or investigate different dosing strategies. There was no restriction on source of BTX-A, dose of administration, injection sites or number of injections.

##### Types of outcome measures

The primary outcome measure for this review was proportion of responders, defined as patients with at least 50% reduction in frequency and/or intensity of pain. For the secondary outcomes of interests, we focused on the mean scores of pain, mean attacks per day and treatment-related AEs.

#### Data extraction and management

A standardized form was used to extract the relevant data on the patients’ and studies’ characteristics, injection protocol, clinical variables, and adverse events by 2 reviewers. Disagreements were resolved by discussion among 3 reviewers.

#### Data interpretation

The extracted data were reviewed, interpreted, and discussed to compile into level data according to “Oxford Center for Evidence-based Medicine” criteria (http://www.cebm.net/index.aspx?o=1025; updated March 2009) for use in clinical practice. The outcome is integrated in the Results and Discussion sections.

### Results

#### Literature search

**Figure 1:**
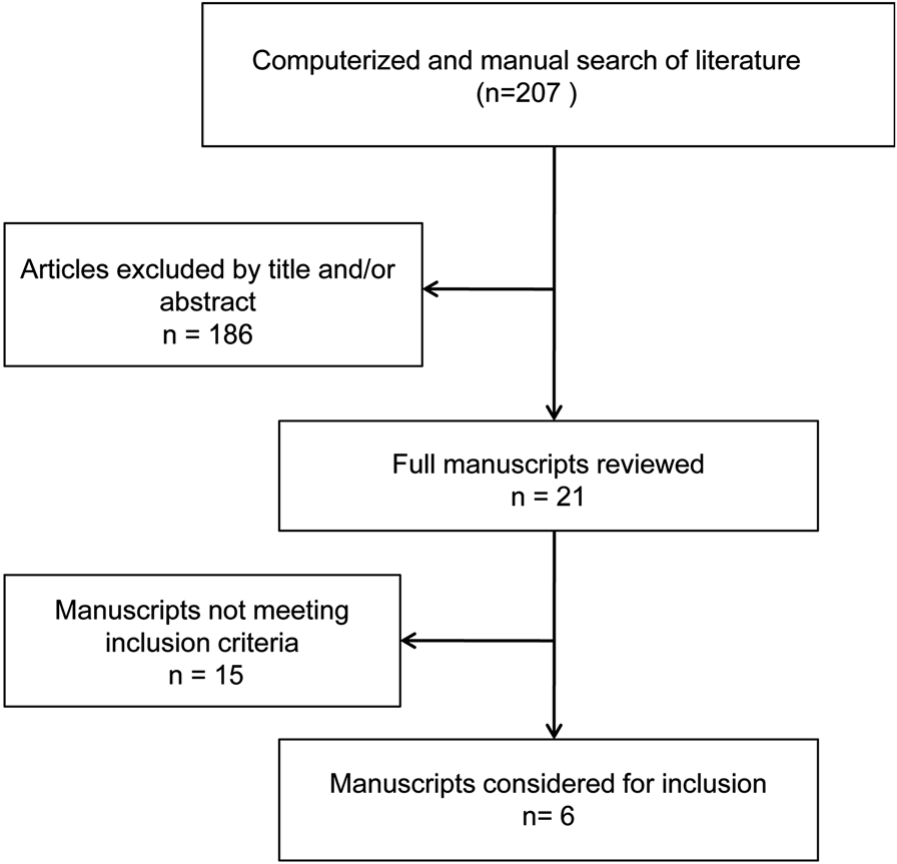
Flow chart illustrating the literature search and evaluation.

Figure 1 gives a flow diagram illustrating the results of the literature search for BTX-A therapy in TN. After a comprehensive search, the references of several review articles were checked, the available studies were evaluated, and then 6 trials [51,56-60] were identified. Two studies of Gazerani et al concerning BTX-A in the treatment of capsaicin-evoked TN were not included in this review [61,62].

#### Study characteristics

Table 1 illustrates the characteristics of studies on the treatment of TN with BTX-A in this review. The number of patients ranged from 8 to 42; and a total of 101 patients were included in 6 selected studies. The majority of studies were open-label studies, except for Wu’s study [56], enrolled 42 patients, in double-blind, randomized and placebo-controlled. Follow-up period ranged from 8 wk to 24 wk, except for a study evaluating the impact of repeated injections which lasted 16-80 wk [60].

**Table 1:** Characteristics of studies and patients for systematic review of BTX-A in the treatment of TN

**Author**	**No. of patients**	**Study design**	**Level of evidence**	**Mean age, year**	**Mean duration before treatment, year**	**Frequency of attacks per day before treatment**	**Pain severity before treatment, VAS**	**Mean follow- up, wk**	**Mean duration of effect, wk**
Wu et al. [56]	42	Randomised, double-blind, placebo-controlled	1b	58.6	5.9	21.2	7.0	12	At least 12
Bohluli et al. [57]	15	Open-label	4	48.9	4.1	33.0	8.0	24	At least 24
Zúñiga et al. [58]	12	Open-label	4	58.5	6.2	23.4^a^	8.8^a^	8	At least 8
Türk et al. [59]	8	Open-label	4	57.1	1.6	unclear	unclear	24	At least 24
Piovesan et al. [51]	13	Open-label	4	61.8	8.8	unclear	9.9	8	At least 8
Borodic et al. [60]	11	Open-label	4	54.2 ^b^	10.0	unclear	unclear	30.6^b^	5-12^b^

^a^ the data of the 10 responded patients.

^b^ the data of all the 44 patients of chronic facial pain.

#### Injection protocol

In most of the studies, the amount of BTX-A injected subcutaneously was 20-50 U in the trigger zones (Table 2). In Wu’s study [56], 75 U of BTX-A (Lanzhou Biological Products Institute) was used in each patient. In addition, 6-9 U and 100 U were used in two independent studies.

**Table 2:** Injection protocol of BTX-A

**Author**	**Source of BTX-A**	**Amount of BTX-A (U)**	**Injection sites**	**No. of injections**
Wu et al. [56]	Lanzhou Biological Products Institute, China	75	Intradermal and/or submucosal trigger zones	15
Bohluli et al. [57]	Unclear	50	Trigger zones	Unclear
Zúñiga et al. [58]	Botox	20-50	Subdermal trigger zones	Unclear
Türk et al. [59]	Botox	100	Region of the zygomatic arch	2
Piovesan et al. [51]	Unclear	6-9	Subdermal trigger zones	Varied for each patient
Borodic et al. [60]	Botox	30-50	Subdermal trigger zones	Unclear

#### Efficacy

##### Primary outcome

**Figure 2:**
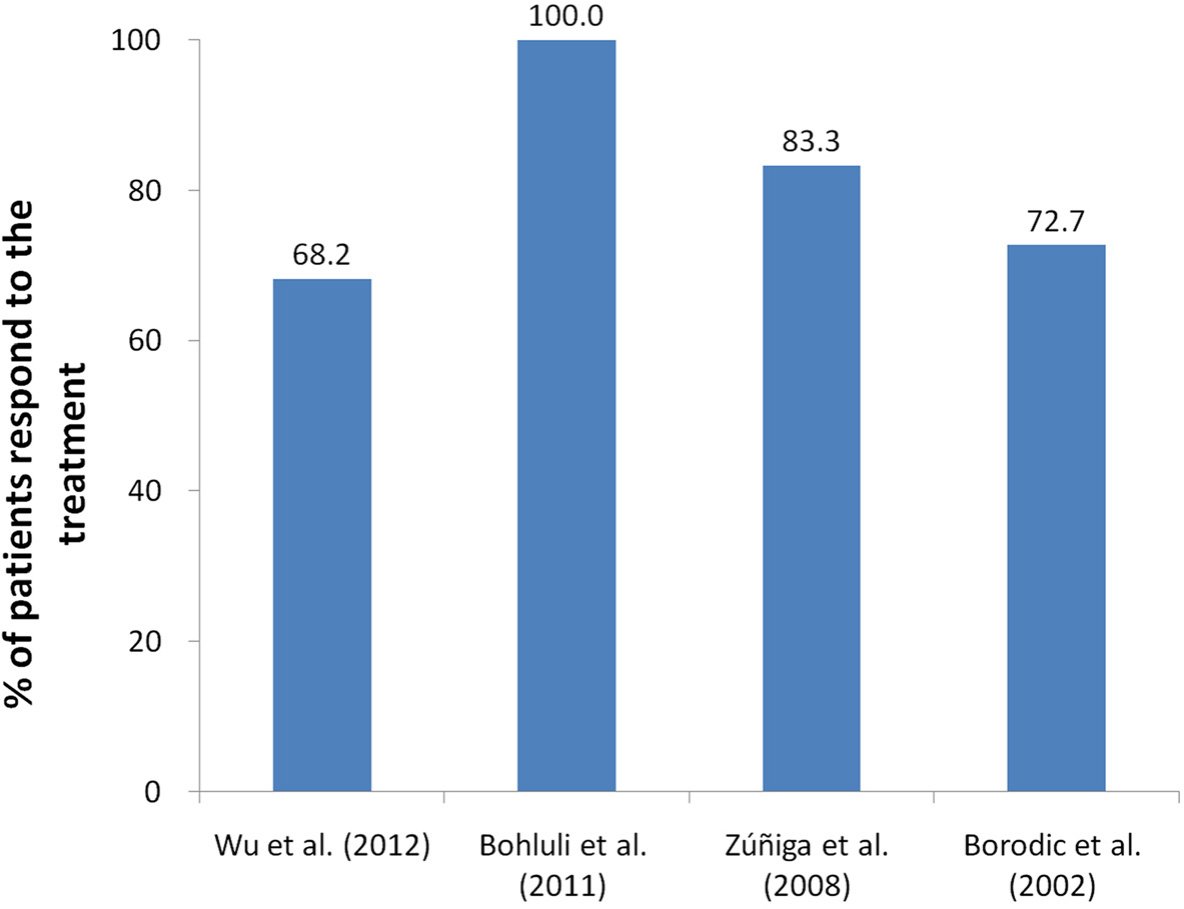
Percent of patients responding to BTX-A treatment.

The proportion of responders, defined as patients with at least 50% reduction in frequency and/or intensity of pain, was all above 60% and the mean proportion was 80% (Figure 2). In Bohluli’s study [57], patients with complete eradication of the pain were also reported: the pain was completely eradicated in 7 patients and there was no need for further medication.

##### Secondary outcomes

In studies reporting the effect of BTX-A on the pain intensity, the mean scores measured by VAS were between 7 and 10 at baseline (Table 3). A controlled study demonstrated that the therapeutic efficacy of BTX-A was significantly superior to that of placebo in pain intensity [56]. Open-label trials confirmed this trend [51,57-60]. After BTX-A injection, the reduction in the mean pain intensity from baseline was 41-81% at 1 wk, 66-98% at 4 wk, about 80% at 8 wk and 12 wk.

**Table 3:** Mean scores of pain measured by VAS

**Author**	**No. of patients**	**Mean baseline (SD)**	**Mean end point (SD)**	**Mean change vs. baseline**	**Mean % change vs. baseline**
Wu et al. [56] placebo					
week 1	20	6.9(2.3)	4.5	2.4	35
week 4	20	6.9(2.3)	4.7	2.2	32
week 8	20	6.9(2.3)	5.1	2.2	26
week 12	20	6.9(2.3)	5.3	1.6	23
Wu et al. [56] BTX-A					
week 1	22	7.1(2.0)	4.2	2.9	41
week 4	22	7.1(2.0)	2.4	4.7	66^a^
week 8	22	7.1(2.0)	1.4	5.7	80^a^
week 12	22	7.1(2.0)	1.4	5.7	80^a^
Bohluli et al. [57]					
week 1	15	8(1.9)	1.5(1.7)	6.5	81^b^
month 1	15	8(1.9)	1.2(1.4)	6.8	85^b^
Türk et al. [59]					
week 1	8	Unclear	Unclear	Unclear	Unclear^b^
month 2	8	Unclear	Unclear	Unclear	Unclear^b^
month 6	8	Unclear	Unclear	Unclear	Unclear^b^
Piovesan et al. [51]^c^					
day 10	13	9.9(0.3)	5.0(3.9)	4.9	49^b^
day 20	13	9.9(0.3)	0.5(2.0)	9.4	95^b^
day 30	13	9.9(0.3)	0.2(1.0)	9.7	98^b^
day 60	13	9.9(0.3)	2.2(2.9)	7.7	78^b^

^a^ p < 0.05 vs placebo.

^b^ p < 0.05 vs baseline.

^c^ mean of three different trigeminal branches.

A controlled study and open-label trials also demonstrated that the therapeutic efficacy of BTX-A was significantly superior to that of placebo in reducing daily pain frequency (Table 4). The mean daily attacks were 21-33 at baseline, but 3.6-8.4 at 1 wk, 4.1-4.7 at 4 wk and 1.8-2.3 at 8-12 wk after BTX-A injection. In Piovesan’s study [51], the average pain area also significantly reduced.

**Table 4:** Mean attacks per day

**Author**	**No. of patients**	**Mean at baseline (SD)**	**Mean at the end (SD)**	**Mean change vs. baseline**	**Mean % change vs. baseline**
Wu et al. [56] placebo					
week 1	20	20.5(10.4)	18.5	2	10
week 4	20	20.5(10.4)	18.8	1.7	8
week 8	20	20.5(10.4)	17.7	2.8	14
week 12	20	20.5(10.4)	18.2	2.3	11
Wu et al. [56] BTX-A					
week 1	22	21.7(22.7)	8.4	13.3	61^a^
week 4	22	21.7(22.7)	4.7	17	78^a^
week 8	22	21.7(22.7)	2.3	19.4	89^a^
week 12	22	21.7(22.7)	1.8	19.9	92^a^
Bohluli et al. [57]					
week 1	15	33.0(18.9)	3.6(5.4)	29.4	89^b^
month 1	15	33.0(18.9)	4.1(5.8)	28.9	88^b^
Türk et al. [59]					
week 1	8	Unclear	Unclear	Unclear	Unclear^b^
month 2	8	Unclear	Unclear	Unclear	Unclear^b^
month 6	8	Unclear	Unclear	Unclear	Unclear^b^

^a^ p < 0.05 vs placebo.

^b^ p < 0.05 vs baseline.

BTX-A was well tolerated in all 6 studies. Although the local or systemic adverse events (AEs) were not very well reported in all studies, most frequent AEs were transient facial asymmetry (Table 5). Facial asymmetry was not severe and resolved within 2 weeks in most studies, except for one patient developing severe side effects which required physiotherapy and took 3 months to resolve in Bohluli’s trial [57]. Other reported AEs of BTX-A injection included transient edema (2.2%), eyelid ptosis (1.1%), dysesthesia (1.1%) and difficulty in chewing (1.1%). Dysphagia and systemic side effects were not reported in all the 5 trials [51,56-60].

**Table 5:** **Treatment-related AEs in the placebo-controlled study of Wu et al**[56]

**Adverse events**	**Placebo**	**BTX-A**
	**(n = 20)**	**(n = 22)**
Transient facial asymmetry	0	5 (23%)
Transient edema	1 (5%)	2 (9%)

#### Analysis of evidence

The evidence for BTX-A in the treatment of TN was quantified as Level 1b on the basis of one properly randomized controlled trial and multiple open-label studies.

### Discussion

From this systematic review, we can conclude that subcutaneous or mucosal injection of BTX-A is effective for adult TN patients.

Response was achieved in approximately 70-100% of patients. In most studies, the mean pain intensity and frequency were reduced by approximately 60-100% at 4 wk after injection. In Bohluli’s study [57], 47% of patients didn’t need further treatment,; nonsteroidal antiinflammatory drugs were enough to alleviate pain in 33% of patients, and 20% of patients again responded to anticonvulsive drugs after BTX-A injection. In Piovesan’s study [51], the pain area was reduced after injection. However, in the majority of studies, changes in medications and pain area throughout the study were not clearly described. A better understanding of this field requires more studies In the future.

BTX-A has a faster onset of action with its significant effect reaching within 1-2 wk and maximum effect within 4-6 wk. Two studies suggest that the effect of a single BTX-A injection could last for 6 mo or approximately 24 wk [57,59], whereas a few studies show the efficacy reduced at 4-8 wk after treatment. The duration that the therapeutic effect continues should be studied in future well designed trials.

Before injection, physicians should adequately inform TN patients about the potential risk of BTX-A-related AEs. Although BTX-A was well tolerated in TN patients, transient facial asymmetry, transient edema, eyelid ptosis, dysesthesia and difficulty in chewing were still reported in 6 studies. To adequately assess the incidence of specific AEs and prevent the underestimate, future studies should adequately document and report the local and systemic AEs.

An important issue is, based on the currently available evidence and physician experience, how BTX-A can be best applied in clinical practice?

The first question is the dosage of BTX-A. The most commonly used dose of BTX-A is 20-75 U. However, Piovesan et al [51] found that 6-9 U of BTX-A induced significant decreases in the pain area and intensity, suggesting that lower doses are also feasible. Türk et al [59] also reported the effectiveness after treatment with 100 U of BTX-A. Because no study was designed to compare the therapeutic efficacy of BTX-A at different doses, the optimal dose cannot be concluded. Also, no study was undertaken to compare of the efficacy or tolerance of BTX-A from different manufacturers.

Another variable is the number of injection sites. In Wu’s study [56], injection was done at 15 sites. However, injection was done at only 2 sites in Türk’s study [59]. It is still unclear if the same efficacy with a less painful and faster injection can be achieved by reducing the number of injections with the same dose of BTX-A.

The optimal indications for re-injection are also important, but they weren’t clarified in these studies. In our opinion, re-injection should be performed only when the worsening of symptoms is present. Patients should not receive repeated injections once the symptomatic improvement occurs after two injections, or severe AEs are present.

## Conclusions

We speculate that BTX-A treatment may provide a clinically significant benefit to TN adults. The effect is rapidly achieved, usually within 1-2 wk. Of importance, BTX-A treatment seems to be well tolerated with minimal injections and to result in limited systemic adverse events. Therefore, it represents a promising treatment of TN with favorable risk-to-benefit ratio. However, well-designed randomized, controlled, double-blinded trial is still lacking. Future adequately powered studies are needed to investigate the optimal dose of BTX-A treatment, the duration of therapeutic efficacy, common AEs, and the time and indications for repeat injection.

## Competing interest

The authors declare that they have no competing interest.

## Authors’ contributions

LJ and YH designed this study. YH, YL and XG carried out the searches, identified studies for inclusion and extracted relevant data. ML, ZN and LF were involved in analysis. LJ acted as arbitrator. All authors read and approved the final version.
